# Nucleophilic Trapping Nitrilimine Generated by Photolysis of Diaryltetrazole in Aqueous Phase

**DOI:** 10.3390/molecules19010306

**Published:** 2013-12-27

**Authors:** Yixin Zhang, Wujun Liu, Zongbao K. Zhao

**Affiliations:** 1 Division of Biotechnology and Dalian National Laboratory for Clean Energy, Dalian Institute of Chemical Physics, CAS, Dalian 116023, China; E-Mails: zhangyx@dicp.ac.cn (Y.Z.); wujunliu@dicp.ac.cn (W.L.); 2 University of Chinese Academy of Sciences, 19A Yuquanlu, Beijing 100049, China

**Keywords:** photolysis, tetrazole, nucleophilic addition, aqueous-phase reaction, nitrilimine, bioorthogonal reaction

## Abstract

Nitrilimine generated by photolysis of diaryltetrazole in aqueous phase under mild conditions was trapped by nucleophiles including amines and thioalcohols. The representative products were characterized, while products with all 20 natural amino acids and a peptide were observed by MALDI-TOF mass spectroscopy. Competitive studies showed that this reaction also occurred in the presence of acrylamide. These results provided new information for understanding the potential side reactions when tetrazole-alkene pairs were used as a bioorthogonal reaction in labeling proteins and related studies in buffered systems.

## 1. Introduction

Nitrilimines, reactive 1,3-dipoles first reported by Huisgen and co-workers [1], have served as versatile intermediates in the construction of a variety of nitrogen-containing compounds. For example, 1,3-dipolar cycloaddition reactions of nitrilimines with alkenes can produce pyrazoles [2,3], while nucleophilic addition of nitrilimines with amines, thiols or hydroxyls can produce triazenes, thiohydrazones or oxyhydrazones, respectively [4,5,6,7]. These triazene or thiahydrazone skeletons have been successfully applied in the preparation of biologically active, heteroatom-containing compounds including triazoles [8], tetrazines [9], thiadiazines [10] and triazepines [11]. Besides these applications in organic synthesis, nitrilimines have been applied in surface modification of polymer materials and bioorthogonal chemistry [12,13,14].

**Scheme 1:**
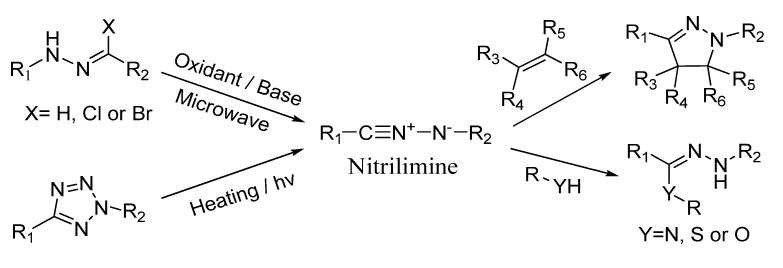
The generation of nitrilimine and related reactions.

A number of methods have been developed to generate nitrilimine. When hydrazones or α-halogenated hydrazones were used as precursors, methods included oxidation [15], microwave heating [16] and treatment with bases [17,18]. When tetrazoles were used, heating [19] and photolysis [20] were effective for nitrilimine formation (Scheme 1). Base-induced dehydrochlorination of hydrazonyl chlorides has also been widely used in organic synthesis. Only a few reports used the photolysis approach, in which tetrazoles were treated by an intense 450 W Hanovia immersion lamp with broad emission spectrum [20,21,22].

Recently, Lin and co-workers reported that nitrilimines could be generated from diaryltetrazoles under mild photoactivation conditions using a hand-held UV lamp of the kind generally used for thin layer chromatography (TLC) monitoring [22,23]. Those nitrilimines were reacted with a variety of alkenyl dipolarophiles for the preparation of pyrazolines. This photoinducible tetrazole-alkene cycloaddition reaction was applied as a bioorthogonal reaction in biological systems. In this study, we explore nucleophilic addition to a diaryltetrazole-derived nitrilimine by amines and thioalcohols in aqueous phase, and the representative products were characterized. Products with all 20 natural amino acids and a peptide were observed by using matrix-assisted laser desorption ionization time-of-flight (MALDI-TOF) mass spectroscopy. Competitive studies showed that this reaction also occurred in the presence of acrylamide. These results provided new information for understanding potential side reactions when tetrazole-alkene pairs are used as a bioorthogonal reaction in labeling proteins and related studies in buffered systems.

## 2. Results and Discussion

### 2.1. Photochemical Reaction between Diaryltetrazole and Nucleophiles

We used 2-(4-methoxyphenyl)-5-phenyltetrazole (**1**) [24] and imidazole as the model system because the tetrazole **1** is more photoreactive and imidazole has high nucleophilicity with only one reactive site. The reaction mixture was irradiated with a hand-held UV lamp at 312 nm for 10 min in a 96-well plate at room temperature in a mixture of CH_3_CN and phosphate buffered saline (PBS). A new spot was identified on the TLC plate. The reaction was scaled up with 0.1 mmol of **1** and 1.0 mmol of imidazole for 1 h, and compound **2** was purified by flash chromatography on silica gel in 34.2% yield (Table 1, entry 1). The structure of **2** was confirmed by ^1^H-, ^13^C-NMR and mass spectra.

**Table 1:** Photochemical reaction between diaryltetrazole and nucleophiles *^a^*. 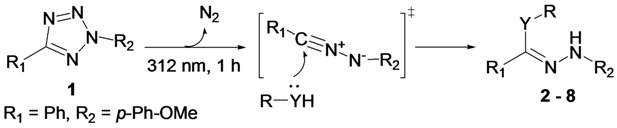

Entry	Nucleophile	Product	Isolated Yield (%)
1	Imidazole	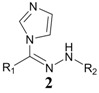	34.2%
2	HOCH_2_CH_2_SH	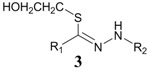	23.3%
3	n-C_4_H_9_NH_2_	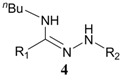	>15% *^b^*
4	Glycine	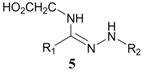	10% *^b^*
5	Histidine	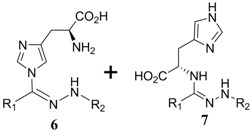	10% *^b^*
6	Phenol	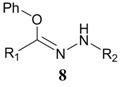	<5% *^b^*

*^a^* The reaction was carried out with tetrazole (0.1 mmol) and nucleophile (1.0 mmol) in 3 mL of CH_3_CN/PBS (v/v, 2:1) under 312 nm irradiation for 1 h; *^b^* Estimated yields by TLC or NMR.

When mercaptoethanol was used as the nucleophile under identical reaction conditions, compound **3** was isolated in 23.3% yield (Table 1, entry 2). Moreover, butylamine, glycine and histidine also acted as nucleophiles and led to the corresponding addition products (Table 1, entries 3−5). In the case of phenol, very little product was observed (Table 1, entry 6). The adduct **4** was unstable and could be partially purified on basic alumina. The product **5** was also unstable. It should be noted that two products, **6** and **7**, were observed when histidine was employed (Table 1, entry 5). These two compounds were separated on TLC plates and showed different colors upon ninhydrin staining. Although high purity was difficult to achieve for compounds **4**−**7**, their structures could be inferred by NMR and mass spectra. It has been known that the conversion of diaryltetrazole to nitrilimine was a fast reaction under UV irradiation, but the nitrilimine intermediate might dimerize [25], or be quenched by water [26,27], resulting in the formation of the addition products in low yields. However, we anticipate that such a mild and convenient photoactivation strategy can be explored as an alternative way for the synthesis of these heterocycles or acyclic compounds upon further process optimization.

### 2.2. Photochemical Reaction between Diaryltetrazole and Amino Acids

We next examined the reactivity of all 20 proteinogenic amino acids with **1** by irradiating the reaction mixtures at 312 nm for 10 min, and new spots were observed by TLC analysis for most of samples. These samples were further analyzed by MALDI-TOF MS. The results shown in Table 2 clearly indicated that nucleophilic addition products were formed.

**Table 2:** MALDI-TOF MS results of reaction products between **1** and amino acids.

Entry	Amino Acid	Coupling Product [M + H^+^]		Entry	Amino Acid	Coupling Product [M + H^+^]	
		Expected	Found			Expected	Found
1	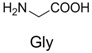	300.0	322.1 (+Na^+^)	11	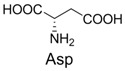	358.1	358.2
2	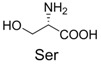	330.0	330.0	12	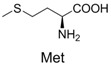	374.2	374.1
3	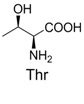	344.1	344.1	13	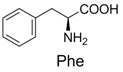	390.1	390.1
4	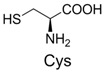	346.1	346.0	14	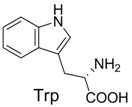	429.2	429.1
5	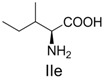	356.1	356.1	15	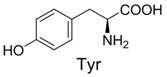	406.1	406.1
6	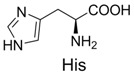	380.1	380.1	16	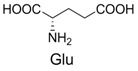	372.1	372.1
7	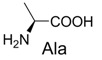	314.1	336.1 (+Na^+^)	17	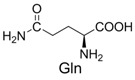	370.1	370.1
8	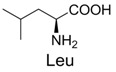	356.1	356.1	18	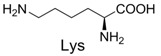	371.1	371.1
9	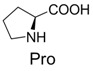	340.1	340.1	19	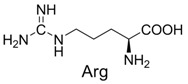	399.2	399.1
10	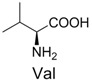	342.1	342.1	20	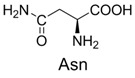	357.1	357.1

### 2.3. Photochemical Reaction between Diaryltetrazole and a Peptide

**Figure 1:**
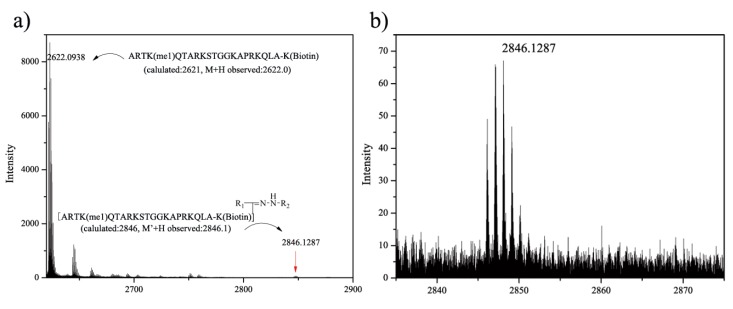
MALDI-TOF MS analysis of photochemical reaction between **1** and peptide. (**a**) MS spectra of the reaction mixture; (**b**) The amplification of the area around 2846 Da.

We also performed the coupling reaction using compound **1** and a peptide. It was clear that a modified peptide was produced, and its MS data had a 224 Da increment compared to that of the parent peptide (Figure 1). No signals as shown in Figure 1b were seen in the control sample experiments.

### 2.4. Competitive Study

To see whether the nucleophilic addition can compete with the 1,3-dipolar cycloaddition reaction, we carried out photochemical reaction between **1** and glycine in the presence of acrylamide.

**Figure 2:**
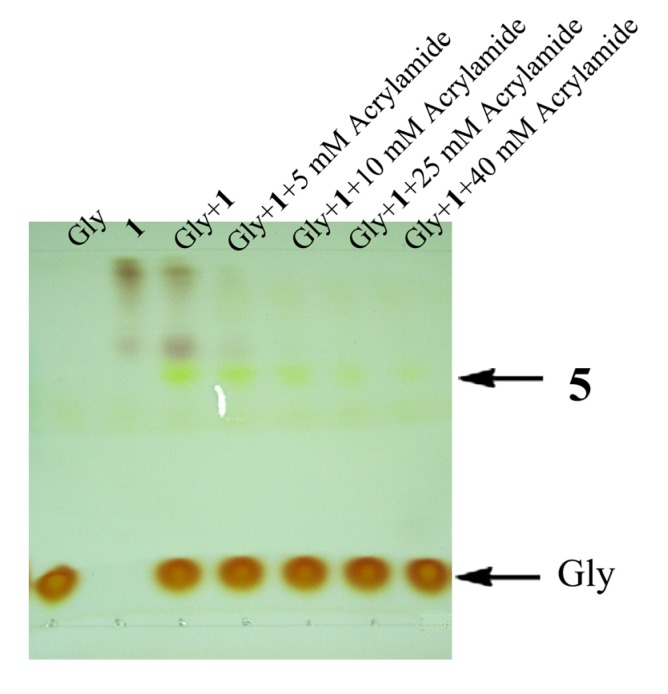
TLC analysis of the photochemical reaction between **1** and glycine in the presence of acrylamide with ninhydrin staining. The reactions were carried out with **1** (25 mM), glycine (25 mM) and acrylamide at different concentrations in CH_3_CN/H_2_O (v/v, 3:1) under UV irradiation at 312 nm for 5 min.

Upon ninhydrin staining, it was observed that the coupling reaction occurred regardless of the present of acrylamide up to 40 mM (Figure 2). Because acrylamide is a relatively reactive dipolarophile, the fact that it failed to suppress the nucleophilic addition suggested that nucleophilic trapping the nitrilimine intermediate in aqueous phase might be more general than expected.

### 2.5. Discussion

Our results showed that the nitrilimine intermediate generated by photolysis of diaryltetrazole in aqueous phase was trapped by a number of nucleophiles, including amino acids and a polypeptide. Interestingly, the nitrilimine intermediate can be trapped more rapidly by alkenes *via* 1,3-dipolar cycloaddition reactions. Based on this reaction, the photoactivated tetrazole-alkene reaction has been applied as a bioorthogonal approach for chemically modifying [28,29,30] and probing protein dynamics and function in live cells [14]. In those examples, proteins were preloaded with a terminal alkene functionality, followed by photoreaction in the presence of excess tetrazoles. For an ideal bioorthogonal reaction, the reaction functionalities must react only with the externally introduced reaction partners, a not with any biological electrophiles and nucleophiles within the cells. As the observation of nucleophilic trapping nitrilimine generated by photolysis of diaryltetrazole in aqueous phase, the bioorthogonality of the tetrazole-alkene approach needs to be reconsidered. Although non-specific labeling of biological samples has not been noticed previously, we recently found that yeast proteome was modified non-specifically when it was irradiated by UV light in the presence of **1** or similar tetrazole compounds [31]. It was also once reported that the nitrilimine intermediate was quenched by H_2_O in the absence of alkene dipolarophiles [26]. This was in agreement with our observations described here. While the current work is not intended to belittle the application of photochemistry of tetrazole in chemo-biological studies, it surely offers alternative interpretation when complicated results are obtained.

## 3. Experimental

### 3.1. General Information

All reagents and chemicals were obtained from commercial suppliers as reagent grade or higher and used without further refinement unless otherwise noted. The polypeptide ARTK(Me1)QTARKSTGGKAPRKQLA-K(Biotin) was generous gift from Prof. Xinmiao Liang of the Dalian Institute of Chemical Physics, Dailan, China. The silica gel used for flash column chromatography was received from Yantai Yuanbo Silica Gel Co., Yantai, China. TLC plates were visualized using a combination of UV, ninhydrin and iodine.

NMR spectra were recorded at room temperature on a Bruker DRX 400 instrument (Bruker Corporation, Fällanden, Switzerland, 400 MHz for ^1^H, 100 MHz for ^13^C). Accurate mass measurements were performed using a Thermo LTQ Orbitrap Elite mass spectrometry (Thermo Fisher Scientific, Waltham, MA, USA) equipped with ESI ionization source. Low resolution mass spectra was recorded on MALDI-TOF/TOF (5,800, AB SCIEX, Framingham, MA, USA), using MALDI with HCCA as matrix for reaction with amino acid, and DHB for reaction with polypeptide. The handheld UV lamp used in the photo click reaction was a Spectroline E-series ultraviolet hand lamp (312 nm, 220 V, Spectronics Corporation, Westbury, NY, USA). The 2-(4-methoxyphenyl)-5-phenyltetrazole was synthesized according to the known procedure [24].

### 3.2. Experimental Procedures and Characterization Data

#### 3.2.1. General Procedure for the Photochemical Reaction between Diaryltetrazole and Nucleophiles

A solution containing tetrazole (25 mg) and nucleophile (10.0 equiv) in CH_3_CN/H_2_O (3 mL, v/v, 2:1) was irradiated with a hand-held 312 nm UV lamp for 1 h. The solvent was then removed *in vacuo* to afford a residue which was subsequently purified by flash chromatography.

*(Z)-1-((2-(4-Methoxyphenyl)hydrazono)(phenyl)methyl)-1H-imidazole* (**2**). Yield 10 mg (34.2%) as a pale yellow solid. ^1^H-NMR (CDCl_3_) δ 7.62 (s, 1H), 7.54 (s, 1H), 7.34−7.41 (m, 6H), 7.11 (d, *J* = 8.8 Hz, 2H), 7.08 (s, 1H), 6.87 (d, *J* = 8.8 Hz, 2H), 3.78 (s, 1H); ^13^C-NMR (CDCl_3_) δ 157.2, 139.9, 139.4, 136.6, 133.3, 131.6, 131.3, 127.5, 121.2, 117.3, 117.2, 105.8. 58.2; HRMS (ESI) calcd. for C_17_H_1__7_N_4_O [M + H]^+^ 293.1358, found 293.1389.

*(Z)-2-Hydroxyethyl N'-4-methoxyphenylbenzohydrazonothioate* (**3**). Yield 7 mg (23.3%) as a yellow solid. ^1^H-NMR (CDCl_3_) δ 7.93 (d, *J* = 7.2 Hz, 2H), 7.41 (t, *J* = 7.4 Hz, 2H), 7.33 (t, *J* = 7.2 Hz, 1H), 7.15 (d, *J* = 6.8 Hz, 2H), 6.89 (d, *J* = 8.8 Hz, 2H), 3.79 (s, 3H), 3.67 (t, *J* = 5.8 Hz, 2H), 2.91 (t, *J* = 6.0 Hz, 2H); ^13^C-NMR (CDCl_3_) δ 157.0, 140.5, 132.2, 131.1, 130.7, 129.4, 117.4, 117.2, 105.8, 64.1, 58.3, 38.0; HRMS (ESI) calcd for C_1__6_H_1__9_N_2_O_2_S [M + H]^+^ 303.1123, found 303.1152.

*(Z)-N-Butyl-N'-(4-methoxyphenyl)benzohydrazonamide* (**4**). The crude residue was purified by basic alumina as a yellow solid. It was unstable after purification. ^1^H-NMR (CDCl_3_) δ 7.55 (d, *J* = 9.6 Hz, 2H), 7.45 (s, 1H, NH), 7.40−7.43 (m, 3H), 6.94 (d, *J* = 8.8 Hz, 2H), 6.82 (d, *J* = 8.8 Hz, 2H), 3.76 (s, 3H), 3.10 (t, *J* = 7.0 Hz, 2H), 1.48 (q, *J* = 5.7 Hz, 2H), 1.33 (s, *J* = 7.5 Hz, 2H), 0.86 (t, *J* = 7.2 Hz, 3H).

#### 3.2.2. General Procedure for the Photochemical Reaction between Diaryltetrazole and Amino Acid

Reactions between tetrazole and 20 different natural amino acids were performed by irradiating a CH_3_CN/PBS mixture (150 μL, v/v, 2:1) of 30 mM tetrazole and an equivalent amount of amino acid in a 96-well plate with a hand-held UV lamp at 312 nm for 10 min. The reaction mixtures were directly analyzed for tetrazole-amino acid coupling by MALDI-TOF MS.

Reaction of glycine with diaryltetrazole was carried out at pH 7.0. Product **5** was soluble in both water and chloroform, but was unstable during purification on silica gel. Tetrazole-Glycine **5**: ^1^H-NMR (D_2_O) δ 7.69 (t, *J* = 7.2 Hz, 2H), 7.57 (dd, *J_1_* = 16 Hz, *J_2_* = 8.4 Hz, 2H), 7.32−7.44 (m, 4H), 6.92−6.97 (m, 3H), 3.73 (s, 3H), 3.72 (s,1H) 3.41 (s, 1H); HRMS (ESI) calcd. for C_16_H_18_N_3_O_3_ [M + H]^+^ 300.1393, found 300.1329.

A solution containing tetrazole (12 mg) and histidine hydrochloride (3.0 equiv) in CH_3_CN/PBS (4 mL, v/v, 1:1, pH 7.0) was irradiated with a hand-held 312 nm UV lamp for 1 h. After the reaction solvent was removed *in vacuo*, the residue was redissolved in water. The soluble part was then separated by a C8 reversed-phase silica gel column to obtain product **7**. The insoluble solid was subsequently dried under reduced pressure, and purified by flash chromatography on silica gel with dichloromethane/methanol (10:1 to 1:1) to give product **6** as a white powder. Tetrazole-Histidine **6**: ^1^H-NMR (CD_3_OD) δ 7.88 (t, *J* = 8.2 Hz, 2H), 7.55−761 (m, 2H)), 7.50 (dd, *J_1_* = 15 Hz, *J_2_* = 7.4 Hz, 2H), 7.32 (d, *J* = 8.4 Hz, 2H), 6.88−6.97 (m, 2H), 6.81 (s, 1H), 3.81 (s, 1.5H), 3.78 (s, 1.5H), 3.05−3.12 (m, 1H), 2.74−2.98 (m, 2H); HRMS (ESI) calcd. for C_20_H_22_N_5_O_3_ [M + H]^+^ 380.1678, found 380.1703. Tetrazole-Histidine **7**: ^1^H-NMR (D_2_O) δ 7.73 (d, *J* = 5.2 Hz, 2H), 7.69 (t, *J* = 8.8 Hz, 2H)), 7.59 (t, *J* = 7.7 Hz, 1H), 7.39−7.46 (m, 2H), 7.27 (d, *J* = 8.8 Hz, 1H), 7.16 (d, *J* = 8.8 Hz, 1H), 6.94 (d, *J* = 9.2 Hz, 2H), 6.90 (s, 1H), 3.74 (s, 1.5H), 3.72 (s, 1.5H), 3.05−3.15 (m, 1H), 2.80−2.95 (m, 2H); HRMS (ESI) calcd. for C_20_H_22_N_5_O_3_ [M + H]^+^ 380.1678, found 380.1705.

#### 3.2.3. General Procedure for the Photochemical Reaction between Diaryltetrazole and Polypeptide

The reaction mixture contained peptide solution (47.5 μL, about 1 mg/mL) and **1** (2.5 μL, 4 mM in DMSO) in water (50 μL). After irradiation with a handheld 312 nm UV lamp for 5 min and incubation at room temperature for 1 h, the reaction mixtures were directly analyzed by MALDI-TOF MS.

## 4. Conclusions

In summary, nitrilimine generated by photolysis of diaryltetrazole can be trapped by nucleophiles such as amines, thioalcohols, amino acids or peptides in an aqueous phase. More attention should be paid to such reactivity profiles in applications when nitrilimine is involved.

## Supplementary Materials

NMR spectra of compounds **2**–**7** are available at: http://www.mdpi.com/1420-3049/19/1/306/s1.
