# Supplementary material for: Nucleophilic Trapping Nitrilimine Generated by Photolysis of Diaryltetrazole in Aqueous Phase
Source: Molecules. 2013 Dec 27;19(1):306–15. doi: 10.3390/molecules19010306 (PMC6271683; doi:10.3390/molecules19010306)

Supporting Information

Figure S1. ^{1}H-NMR spectra of **2** in $CDCl_3$.

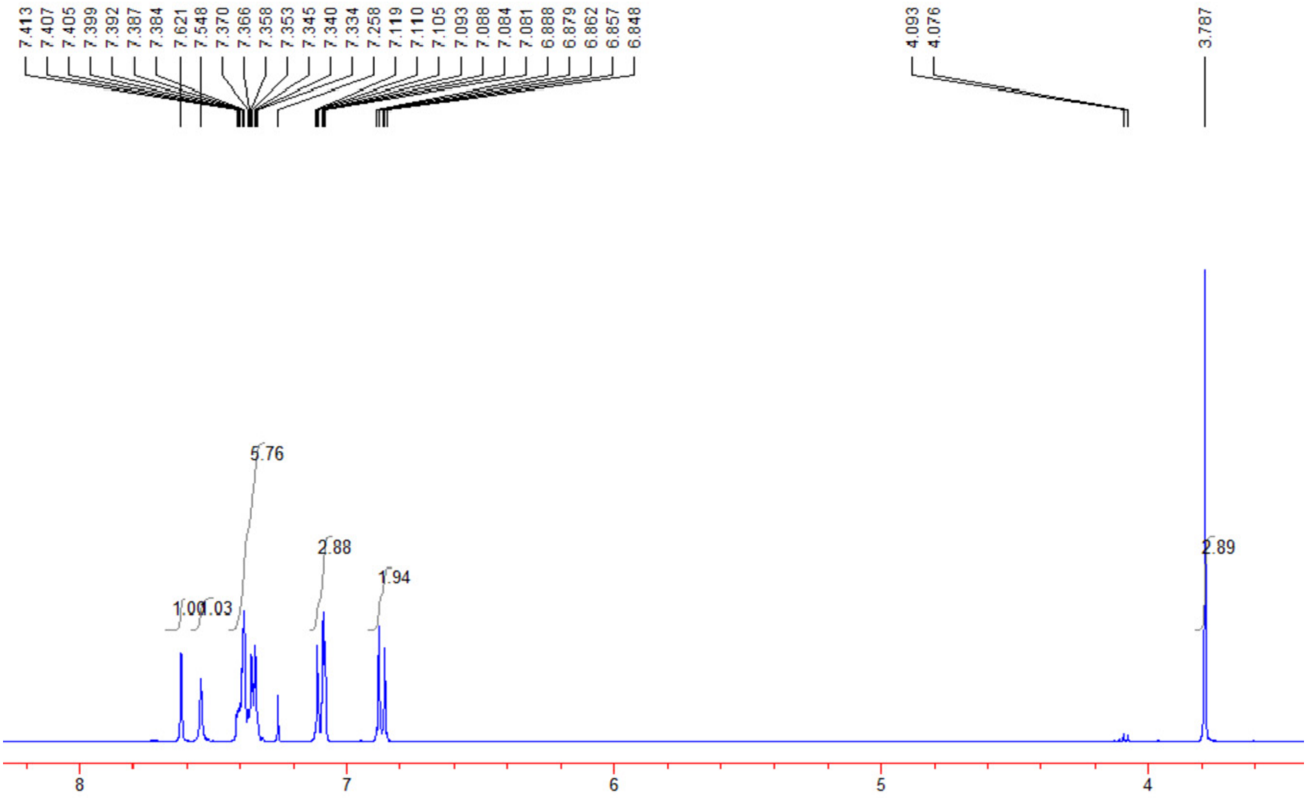

Figure S2. ^{13}C-NMR spectra of **2** in $CDCl_3$.

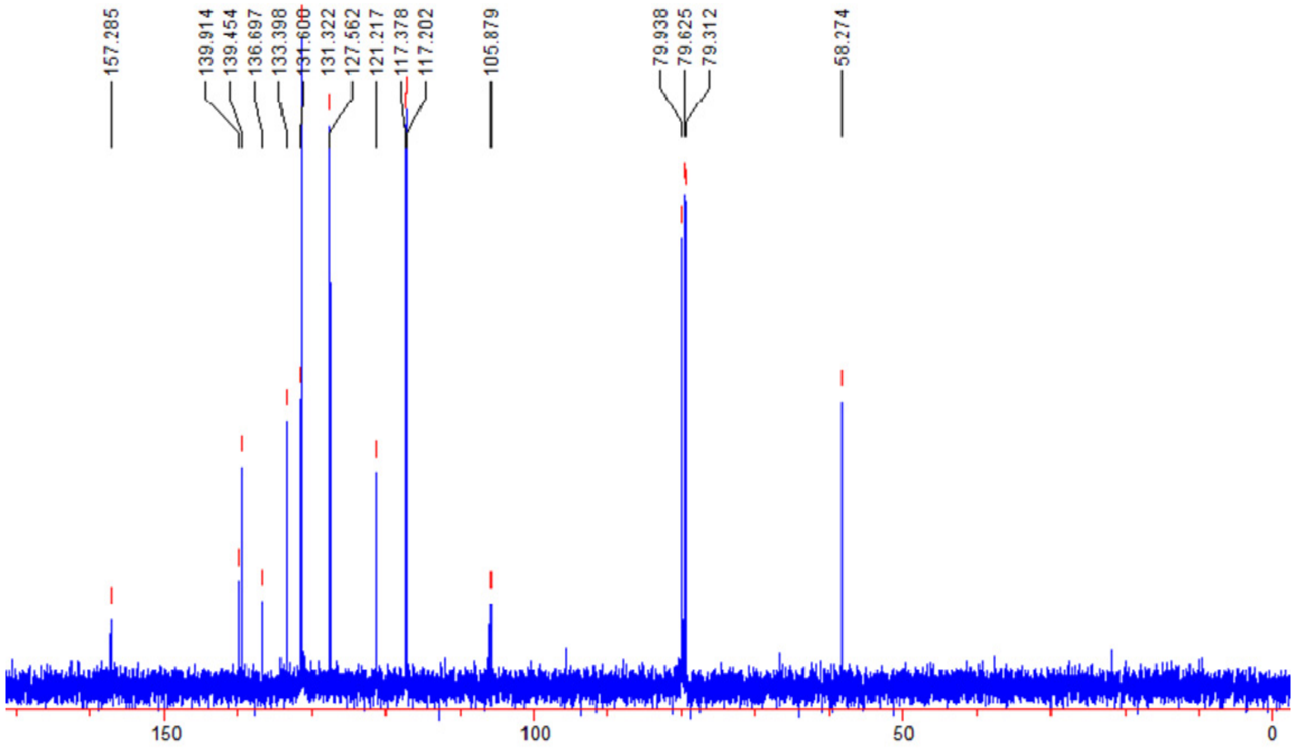

Figure S3. ^{1}H-NMR spectra of **3** in $CDCl_3$.

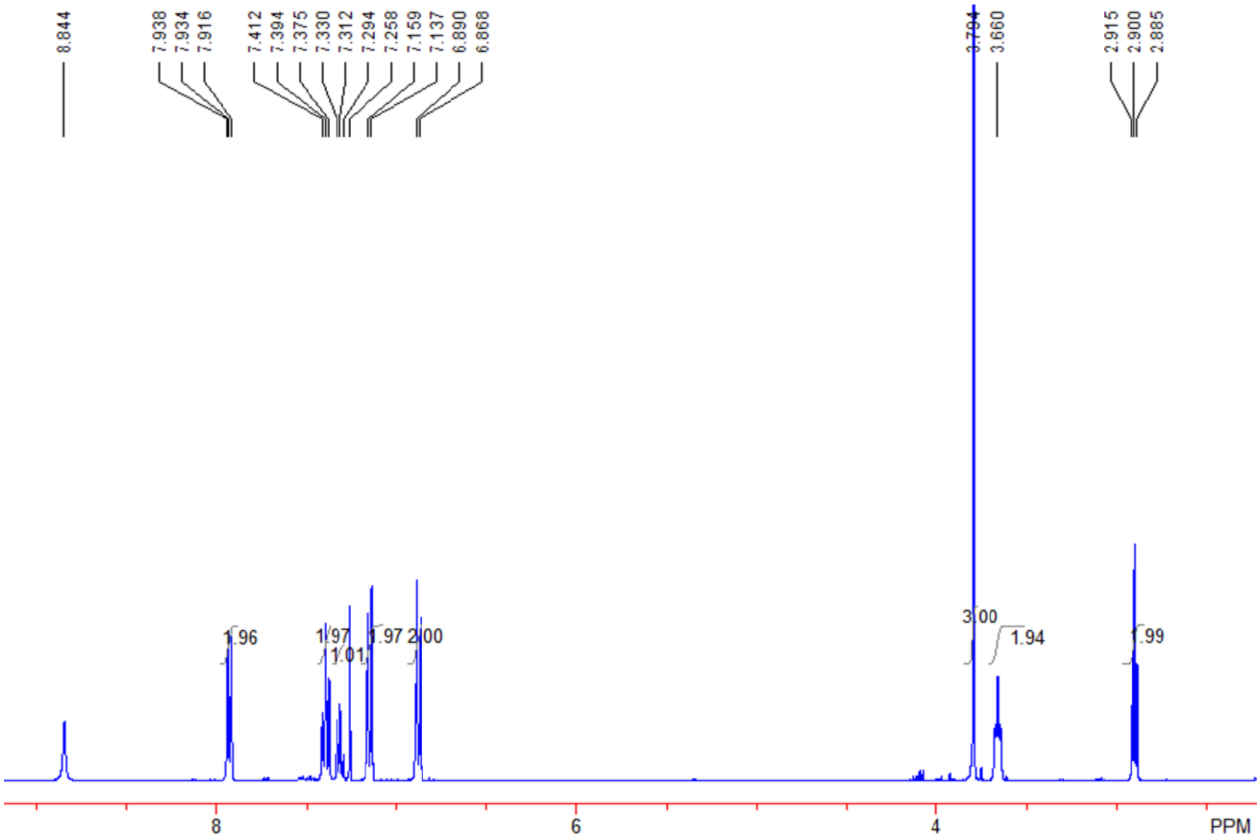

Figure S4. ^{13}C-NMR spectra of **3** in $CDCl_3$.

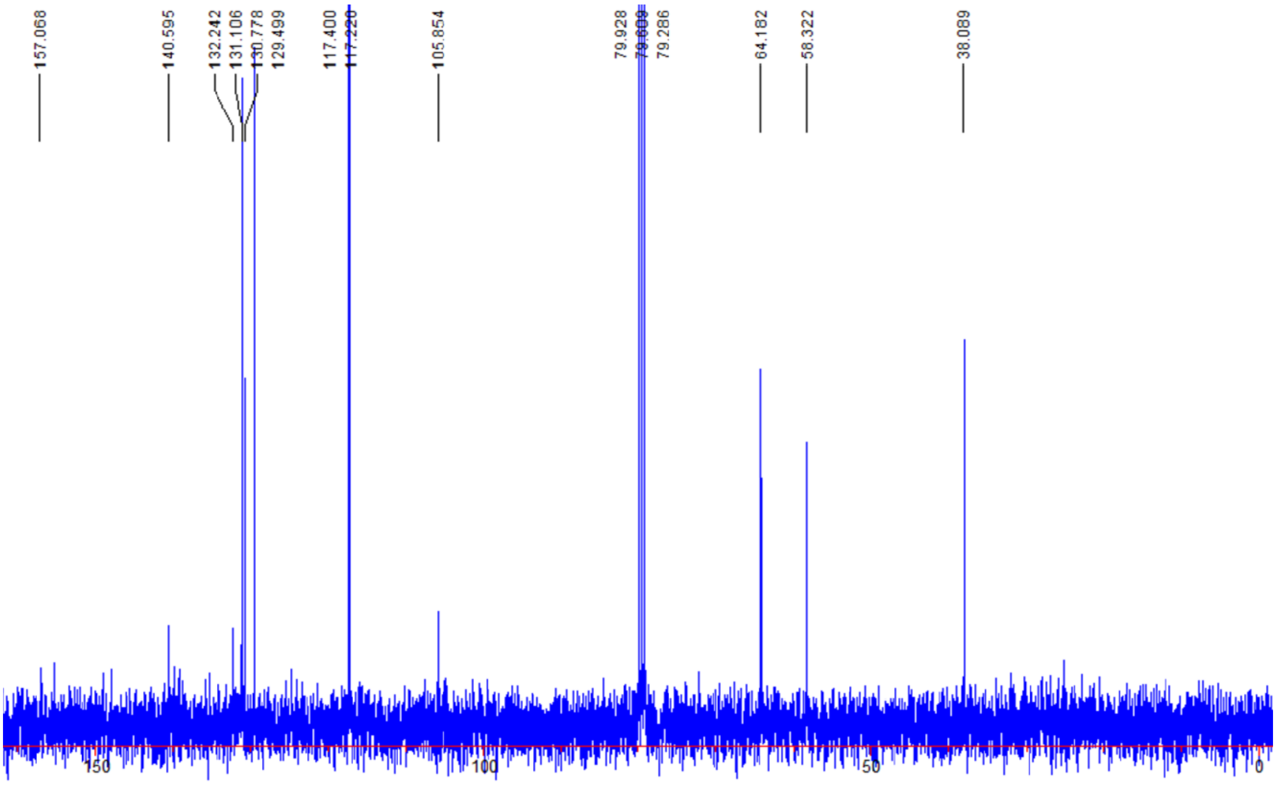

Figure S5. ^{1}H-NMR spectra of **4** in $CDCl_3$.

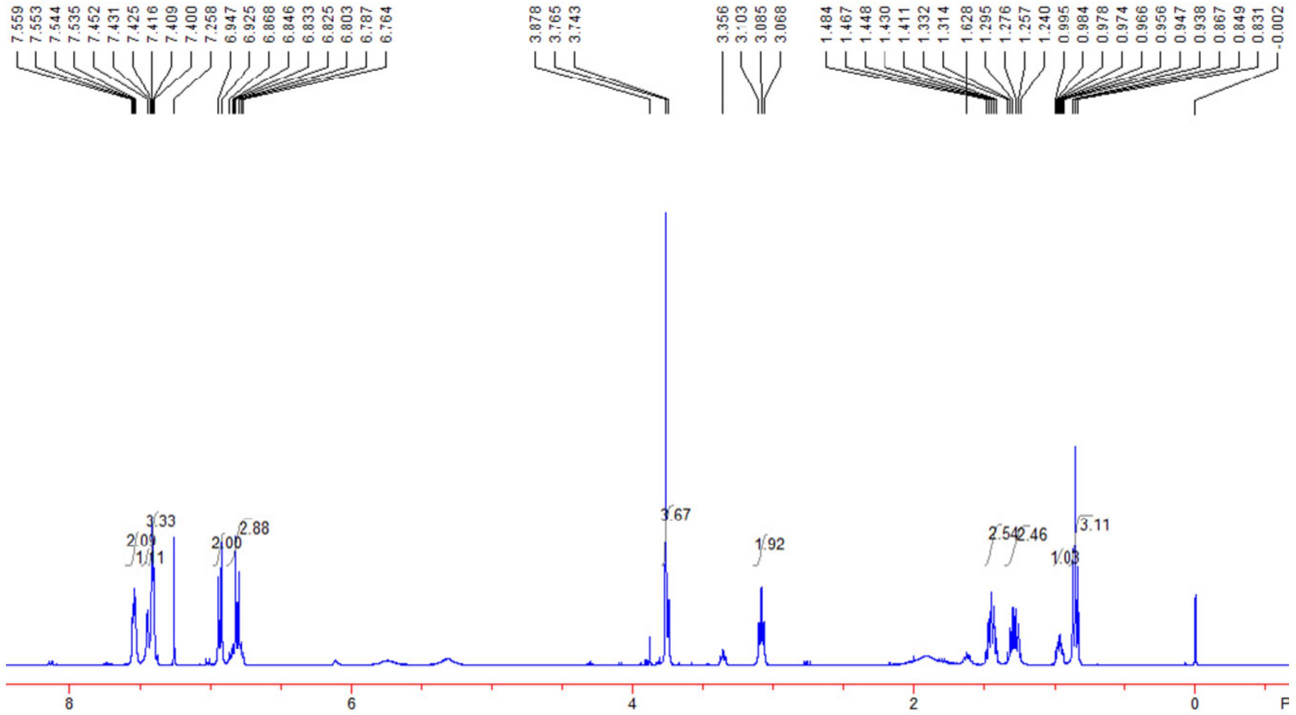

Figure S6. ^{1}H-NMR spectra of **5** in D_2O.

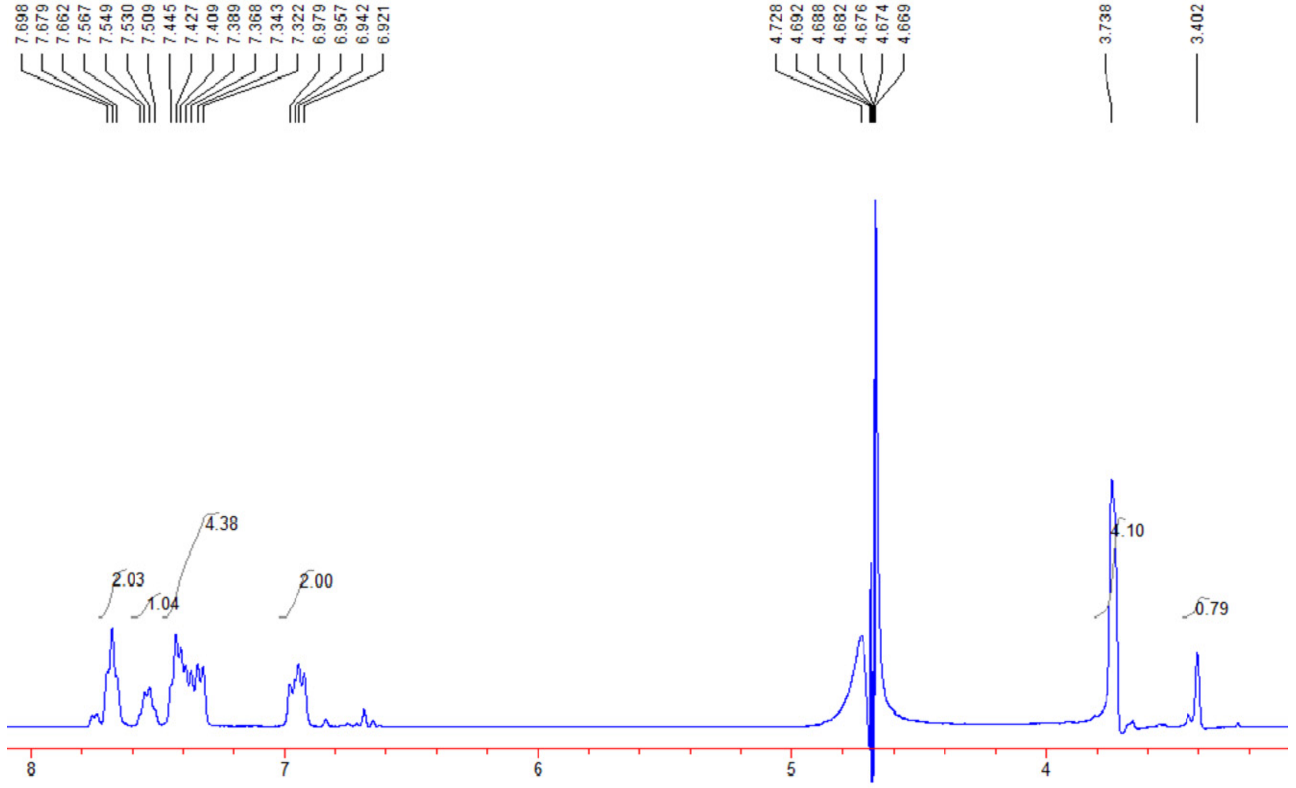

Figure S7. ^{1}H-NMR spectra of **6** in CD_3OD.

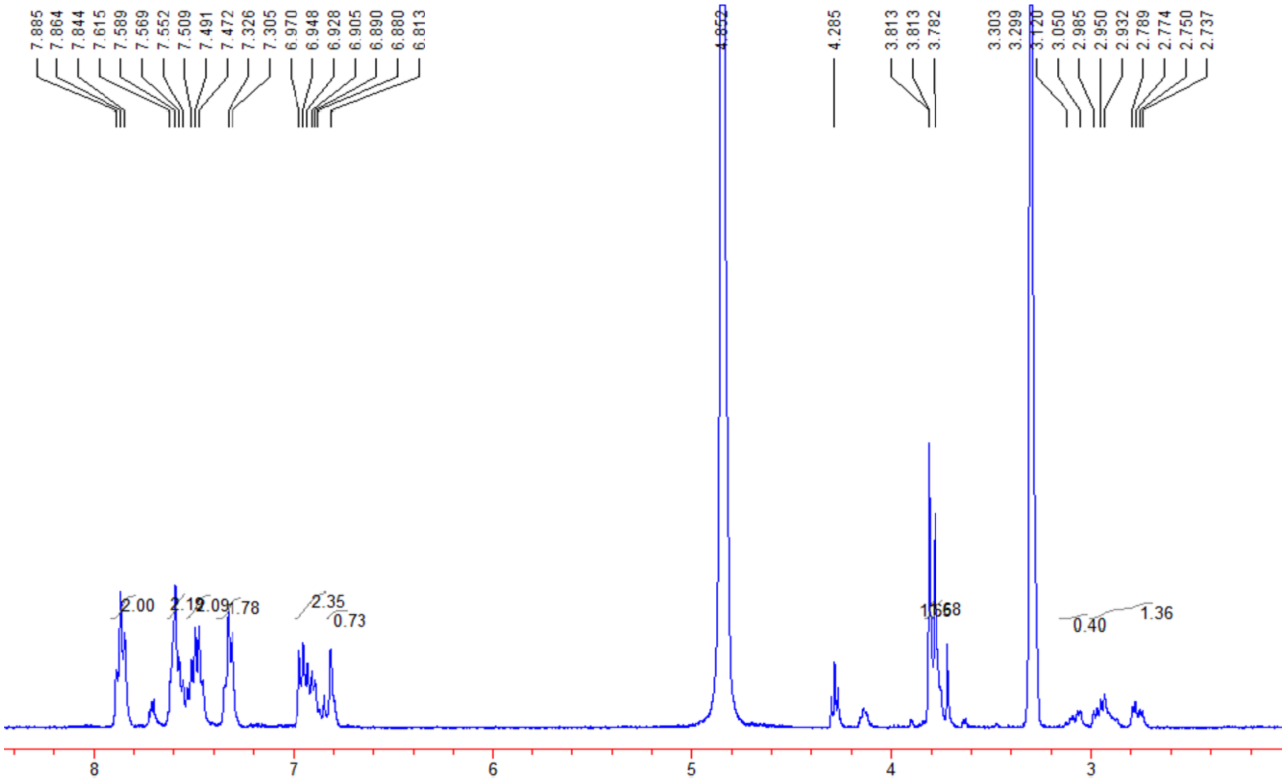

Figure S8. ^{1}H-NMR spectra of **7** in D_2O.

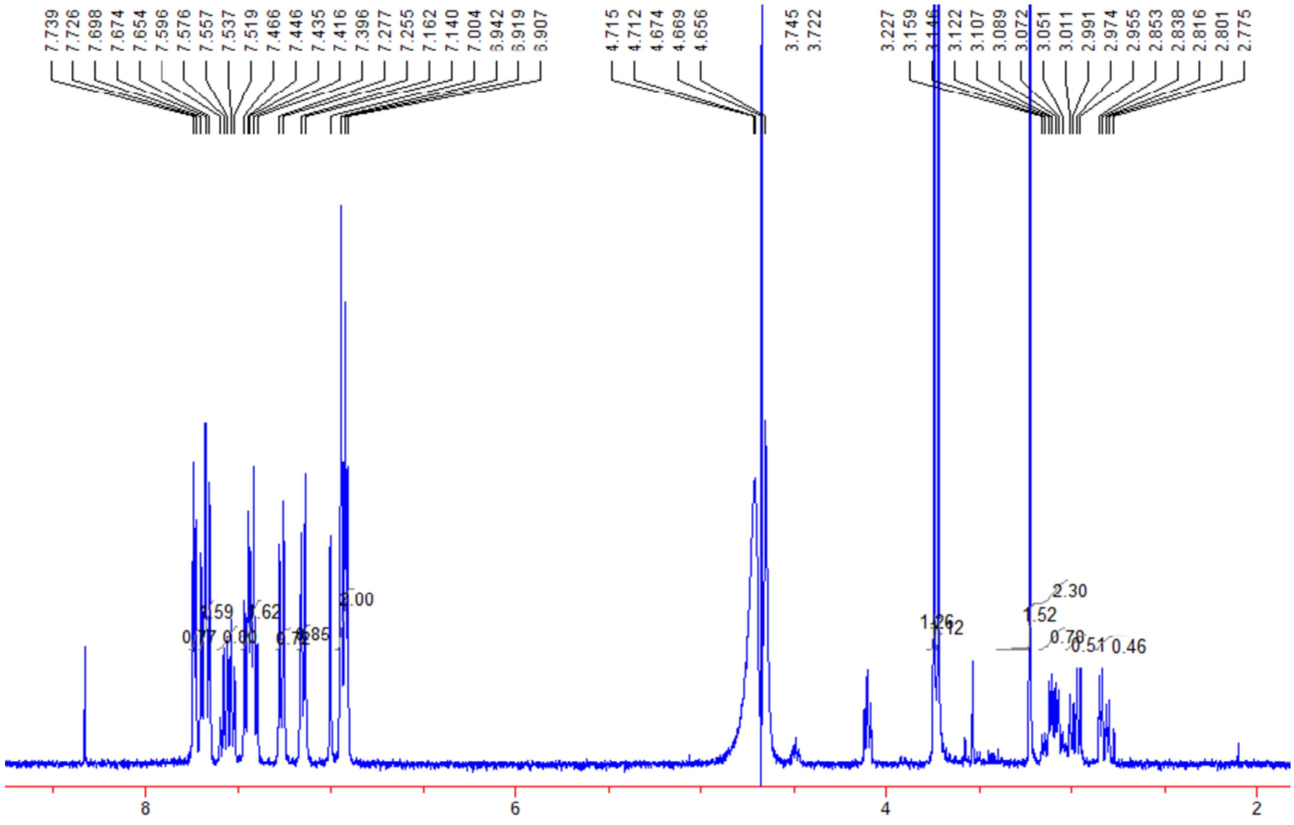

Supplement: Supplementary file 1 [file molecules-19-00306-s001.pdf]
